# Atypical presentation of adenosquamous carcinoma: A case report

**DOI:** 10.1177/2050313X18801217

**Published:** 2018-12-10

**Authors:** Annie Genois, Catherine Maari, Danielle Bouffard

**Affiliations:** 1 Division of Dermatology, Université de Montréal, Montréal, QC, Canada; 2 Department of Pathology and Cellular Biology, Université de Montréal, Montréal, QC, Canada

**Keywords:** Adenosquamous carcinoma, aggressive, squamous cell

## Abstract

Cutaneous adenosquamous carcinoma is a rare malignant neoplasm that is more aggressive than conventional squamous cell carcinoma. The typical clinical presentation is an indurated papule or plaque on the head and neck of elderly patients. The authors report the case of a 52-year-old man with a right scrotal and inguinal tumour measuring 10 cm × 15 cm that had progressed over the past 2 years. The histological examination was compatible with adenosquamous carcinoma. Metastatic inguinal and pelvic lymph nodes were identified. This case demonstrates an atypical presentation of a rare tumour. Adenosquamous carcinoma is more aggressive than conventional squamous cell carcinoma, and prompt diagnosis is important.

## Introduction

Cutaneous adenosquamous carcinoma is a rare malignant neoplasm that is more aggressive than conventional squamous cell carcinoma. The typical clinical presentation is an indurated papule or plaque on the head and neck of elderly patients. This case demonstrates an atypical presentation of a rare tumour.

## Case report

A 52-year-old man presented to the clinic with a right scrotal and inguinal tumour that had progressed over the past 2 years. The patient had no other medical conditions.

On physical exam, the patient had a 10 cm × 15 cm warty and friable scrotal mass. He had two fixed voluminous inguinal lymph nodes bilaterally.

An abdominal and pelvic computed tomographic (CT) scan, a pelvic magnetic resonance imaging (MRI) and a positron emission tomography (PET) scan were performed. The PET scan revealed increased uptake of the inguino-scrotal mass. Metastatic inguinal and pelvic lymph nodes were identified, the biggest measuring 7 cm on the left inguinal side. There were no other primary lesions noted. There was no distant metastasis.

Many biopsies were taken from the cutaneous inguinal mass and revealed a dense proliferation of atypical epithelial cells in the dermis extending and ulcerating the epidermis. Numerous mitotic figures and apoptotic cells were observed as well as focal squamous and glandular differentiation. Immunohistochemistry was performed and the tumour was diffusely positive for CK5/6 and CK AE1/AE3. It was also positive for p63 except in the cells bordering the lumen of the glands. GATA3 was positive in a proportion of the tumour cells.

Initially, our differential diagnosis included a malignant adnexal carcinoma and an adenosquamous carcinoma. Moreover, because of GATA3 positivity, a urothelial origin could not be excluded. Although an adnexal malignant carcinoma cannot be entirely excluded since the whole tumour was not examined pathologically for a residual benign component, the clinical history is negative for transformation of a long-standing tumour. Finally, a urothelial origin was less probable in the light of a negative radiology workup.

The patient was not immunosuppressed and was seronegative for HIV. The patient’s diagnosis was a primary cutaneous adenosquamous cell carcinoma with regional lymph node metastasis.

The initial treatment plan consisted of neoadjuvant chemotherapy with 15 cycles of cisplatin 5-FU followed by surgical excision. The tumour regressed after three cycles of chemotherapy but subsequently started progressing again. Given this evolution, the patient was referred for palliative radiotherapy.

## Discussion

Cutaneous adenosquamous carcinoma is an extremely rare malignant neoplasm that was first described in 1985.^1^

The typical clinical presentation is an erythematous and indurated papule or plaque with a predilection for the head, neck or upper extremities in a person with a history of actinic damage. It tends to affect elderly or immunocompromised individuals.^2^ In the largest case series of adenosquamous carcinoma, the key epidemiologic features of 27 patients were evaluated.^2^ The mean age was 74 years. The tumours were located on the face and scalp in 70% of the patients and on the upper extremities in 15% of the patients. No metastases occurred in this case series.

Locoregional recurrence is frequent, so patients must be monitored closely. Local recurrence rates are between 22% and 26%,^3^ but distant metastasis is rare.^4^ Important predictors of extensive local disease and recurrence include depth of invasion, microscopic perineural invasion and immunosuppression.^2^

Not only is cutaneous adenosquamous carcinoma an extremely rare entity but its presentation was highly atypical in our patient. First, the patient was not elderly nor immunosuppressed. Second, the lesion occurred on the lower extremity rather than the upper extremity. Third, the patient had lymph node metastases.

**Figure 1.**
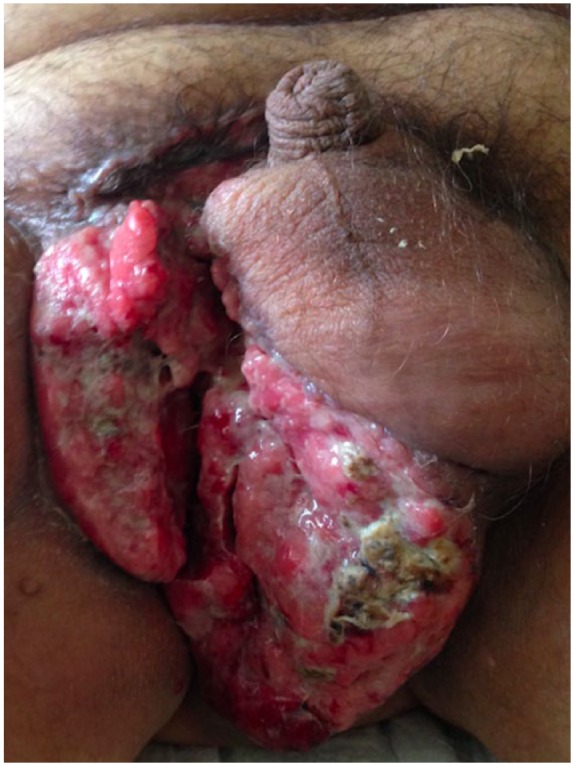
Clinical presentation of adenosquamous carcinoma. Large fungating mass located in the inguinal and scrotal area.

**Figure 2.**
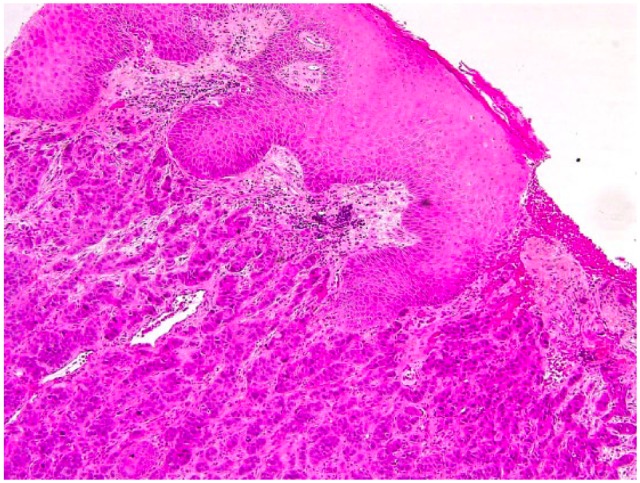
Histopathology of adenosquamous carcinoma. Stain: hematoxylin phloxine saffron (HSP). Magnification: ×10. Tumour extending and ulcerating the epidermis.

**Figure 3.**
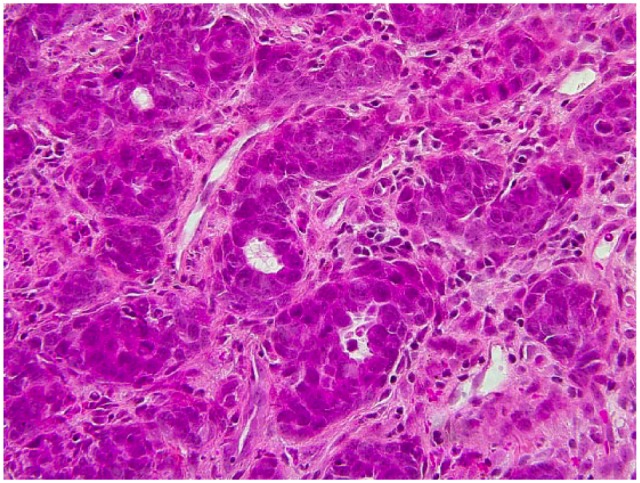
Histopathology of adenosquamous carcinoma. Stain: HSP. Magnification: ×40. Close-up of glandural differentiation.

**Figure 4.**
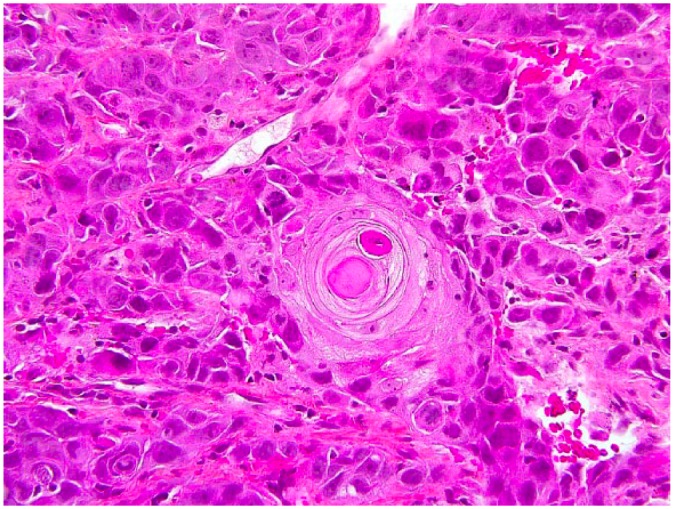
Histopathology of adenosquamous carcinoma. Stain: HSP. Magnification: ×40. Close-up of squamous differentiation.

Our case is consistent with the fact that adenosquamous carcinoma is more aggressive than conventional squamous cell carcinoma. Given its rarity, data are sparse regarding its epidemiology and rates of recurrence and metastasis. This case highlights the fact that this entity can present with a different clinical presentation than the one previously described. Prompt diagnosis is important in order to treat this aggressive tumour (Figures 1–4).
